# Multi-scale mechanobiological model for skeletal muscle hypertrophy

**DOI:** 10.3389/fphys.2022.899784

**Published:** 2022-10-06

**Authors:** Yesid Villota-Narvaez, Diego A. Garzón-Alvarado, Oliver Röhrle, Angelica M. Ramírez-Martínez

**Affiliations:** ^1^ Numerical Methods and Modeling Research Group (GNUM), Universidad Nacional de Colombia, Bogotá, Colombia; ^2^ Institute for Modelling and Simulation of Biomechanical Systems, University of Stuttgart, Stuttgart, Germany; ^3^ Biomimetics Laboratory, Instituto de Biotecnología (IBUN), Universidad Nacional de Colombia, Bogotá, Colombia; ^4^ Stuttgart Center for Simulation Sciences (SC SimTech), Stuttgart, Germany; ^5^ Biomedical Engineering Department, Engineering Faculty, Universidad Militar Nueva Granada, Bogotá, Colombia

**Keywords:** dynamical systems, population dynamics, cellular signaling pathways, biochemical modeling, muscle adaptation, mechanobiology, biomechanics

## Abstract

Skeletal muscle adaptation is correlated to training exercise by triggering different signaling pathways that target many functions; in particular, the IGF1-AKT pathway controls protein synthesis and degradation. These two functions regulate the adaptation in size and strength of muscles. Computational models for muscle adaptation have focused on: the biochemical description of signaling pathways or the mechanical description of muscle function at organ scale; however, an interrelation between these two models should be considered to understand how an adaptation in muscle size affects the protein synthesis rate. In this research, a dynamical model for the IGF1-AKT signaling pathway is linked to a continuum-mechanical model describing the active and passive mechanical response of a muscle; this model is used to study the impact of the adaptive muscle geometry on the protein synthesis at the fiber scale. This new computational model links the signaling pathway to the mechanical response by introducing a growth tensor, and links the mechanical response to the signaling pathway through the evolution of the protein synthesis rate. The predicted increase in cross sectional area (CSA) due to an 8 weeks training protocol excellently agreed with experimental data. Further, our results show that muscle growth rate decreases, if the correlation between protein synthesis and CSA is negative. The outcome of this study suggests that multi-scale models coupling continuum mechanical properties and molecular functions may improve muscular therapies and training protocols.

## 1 Introduction

Training exercise has an important effect on skeletal muscle anatomy and physiology by means of increasing the protein content. Whereas training exercise is related to the mechanical behavior of muscle, increasing protein content is related to its biological behavior.

From the biological perspective, protein synthesis is promoted by the IGF1-AKT signaling pathway (Sandri, 2008; Schiaffino and Mammucari, 2011). The most prominent relation in this pathway starts when insuline-like growth factor (IGF1) (Adams and McCue, 1998) promotes the serine/threonine kinase (AKT) (Song et al., 2005). After that, AKT promotes the mammalian target of rapamycin (mTOR) (Hay and Sonenberg, 2004) and inhibits the forkhead box transcription factor (FOXO) (Wang et al., 2014). Two outcomes can occur: hypertrophy or atrophy. If FOXO is inhibited, then mTOR activates protein synthesis to produce hypertrophy. If AKT is inhibited, then FOXO promotes protein degradation and the inhibition of mTOR resulting in muscle atrophy.

The regulation of protein content is the key to preserve or improve the ability to generate force. On a subcellular scale, force generation is closely related to its microstructure and can be explained by the sliding filament theory (Hanson and Huxley, 1953; Pollack, 1983; Huxley, 2000) and the cross-bridge theory (Huxley, 1957). According to the sliding filament theory, internal sarcomere constituents -actin and myosin- overlap and change the overlapping length while momentarily bound to one another; this short-lasting bound generates force and is known as cross-bridge (Huxley and Hanson, 1959; Randall et al., 2002; Gautel, 2008; Rui et al., 2010). The force generation process starts when the nervous system sends electrical signals to muscle fibers triggering a muscle contraction (Matthews, 1931; Kraus et al., 1994; De Deyne, 2001).

From a mechanical perspective, one can distinguish between a passive and an active response; the active response accounts for muscle contraction. The force generated during contraction is assumed to be a function of the fiber stretch that occurs during the actin and myosin overlap (Gordon et al., 1966; Flitney and Hirst, 1978; Stephenson and Williams, 1982). In contrast, a passive response occurs when a muscle is stretched without contracting.

In a continuum-mechanical setting, the active (Ramírez et al., 2010; Heidlauf et al., 2017) and passive (Takaza et al., 2013; Bleiler et al., 2019) responses of skeletal muscle tissue are modelled by appealing to hyperelastic transversely isotropic constitutive laws. Growth has been considered within continuum-mechanical models of cardiac tissue and to describe the longitudinal growth of skeletal muscles (Göktepe et al., 2010; Zöllner et al., 2012; Altan et al., 2016). Growth is typically based on a multiplicative decomposition (Rodriguez et al., 1994) of the deformation gradient into elastic and growth components. The elastic component requires the characteristic material response of the tissue (passive response), whereas the growth component requires further assumptions like growth multipliers (Göktepe et al., 2010). The continuum mechanical description of growth is a macroscopic and purely phenomenological process and lacks direct links to the underlying biochemical aspects.

Growth is a biochemical response that requires weeks to produce an increase in protein content, and is triggered by an intermittent but regular stimulus (training or physical activity). Although existing continuum-mechanical models of muscle tissue growth predict adaptation in the scale of weeks, those models require continuous stimulation (sustained strain or stress), and none of them considers biochemical processes to predict the resulting change in mass.

Given a training protocol, a model that predicts changes in muscle properties greatly benefits high performance athletes, recovery patients after injuries or illnesses, and the general public. Such model allows to fine tune the protocol (training frequency, intensity, physical restrictions etc) to efficiently target desired outcomes. The aim of this work is to present a multi-scale mechanobiological model for skeletal muscle adaptation. After briefly introducing the independent models for the IGF1-AKT signaling pathway (biochemical model) and fundamental equations of continuum-mechanical basis, i.e., how to model active and passive responses for muscle tissue (mechanical model), we propose a novel description to 1) couple the biochemical model to the mechanical model (by defining of a growth multiplier); 2) couple the mechanical model to the biochemical model (by defining an appropriate feedback function). To validate our new model, we predict the muscular changes due to a specific training protocol and compare the outcome with experimental data. We conclude by discussing our findings.

## 2 Materials and methods

This section is divided into three parts: Section 2.1 (Independent models) describes the main characteristics of the IGF1-AKT signaling pathway model and the skeletal muscle passive and active hyperelastic material model; Section 2.2 (Mechanobiological model) presents the procedures to couple: first, the biochemical to the mechanical model, and second, the mechanical to the biochemical model; Section 2.3 (Numerical experiments) provides details for the computational implementation, the finite-elements structure, the exercise-training protocol, and the biochemical model parameters.

### 2.1 Independent models

The independent models are introduced in this section. The biochemical model in Section 2.1.1 describes the dynamical system that explains the interaction between the variables of the IGF1-AKT signaling pathway. The mechanical model in Section 2.1.2 describes the characteristics of the continuum model for skeletal muscle mechanical response.

#### 2.1.1 Biochemical model

The IGF1-AKT signaling pathway is triggered by an external stimulus; as a result, the concentration of each molecule involved in this pathway evolves producing hypertrophy or atrophy. Based on the simplified mechanism of the IGF1-AKT signaling pathway reviewed by Schiaffino and Mammucari (2011), we developed the biochemical model presented in Villota-Narvaez et al. (2021). Our biochemical model produces the evolution of the cellular myofibril content, and it is suitable for atrophy scenarios, whereas the model presented in the present paper improves the hypertrophy results. We briefly summarize our biochemical model in this section.

We assume that the essential molecules of the pathway are IGF1, AKT, FOXO, and mTOR, and each molecule is represented by variables *x*_1_, *x*_2_, *x*_3_, and *x*_4_ respectively. The variables are treated as populations that interact in a Lotka-Volterra system.

**FIGURE 1:**
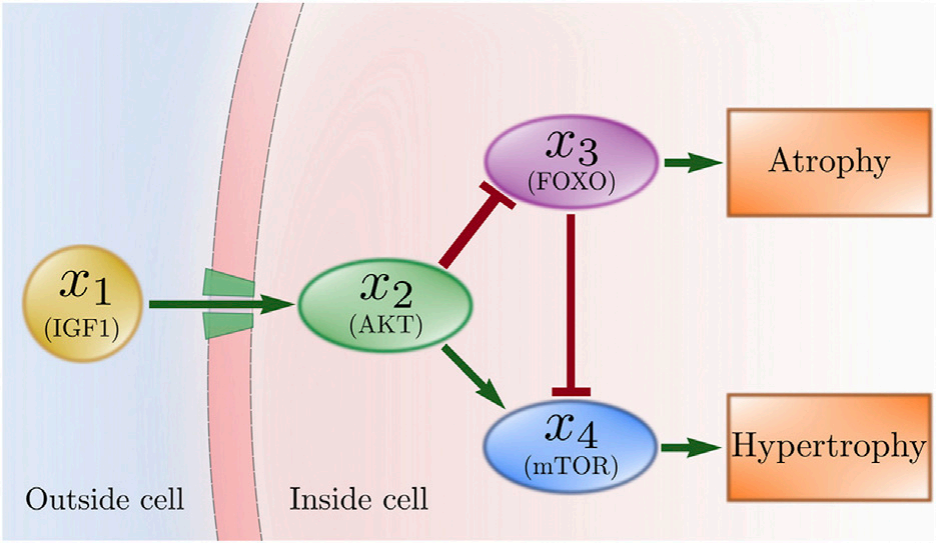
Simplified signaling pathway for muscle adaptation presented by Schiaffino and Mammucari (2011). IGF1 activates AKT, AKT activates mTOR and inhibits FOXO, FOXO inhibits mTOR and promotes atrophy, mTOR promotes hypertrophy.

Following the pathway in Figure 1, physical activity produces an increase in *x*_1_ that remains high as long as the activity is sustained. The increase in *x*_1_ produces an increase in *x*_2_ that persists for about 6 h after the activity is stopped (Bickel et al., 2005). The increase in *x*_2_ produces both an increase in *x*_4_ and a decrease in *x*_3_. An increased *x*_4_ correlates to increased protein synthesis and, in the long term, to hypertrophy. In contrast, drastically reduced physical activity produces a decrease in *x*_1_. The decrease in *x*_1_ produces a decrease in *x*_2_. The decrease in *x*_2_ causes both a decrease in *x*_4_ and an increase in *x*_3_. An increased *x*_3_ correlates to protein degradation and, in the long term, to atrophy. Figure 1 sketches the interaction between these variables.

The coupled ordinary differential equation system is

$${\dot{x}}_{1}=x_{1}\;\left(\;a_{1}\left(t\right)-\;b_{1}\;x_{1}\;\right)$$ (1a)

$$\begin{array}{ll} {\dot{x}}_{2} & =x_{2}\;\left(\;a_{2}\left(t\right)-\;b_{2}\;x_{2}+\;c_{21}\;x_{1}\;\right) \end{array}$$ (1b)

$$\begin{array}{ll} {\dot{x}}_{3} & =x_{3}\;\left(\;a_{3}-\;b_{3}\;x_{3}-\;c_{32}\;x_{2}\;\right) \end{array}$$ (1c)

$$\begin{array}{ll} {\dot{x}}_{4} & =x_{4}\;\left(\;a_{4}-\;b_{4}\;x_{4}+\;c_{42}\;x_{2}-\;c_{43}\;x_{3}\;\right) \end{array}$$ (1d)

$$\begin{array}{ll} \dot{z} & =f\left(x_{3},x_{4}\right). \end{array}$$ (1e)

Terms in Eqs 1a, 1b, 1c, 1d, 1e are: *x*_*i*_ is the population of molecule *i*, coefficient *a*_*i*_ is the intrinsic growth rate of the molecule *i*, and coefficient *c*_*ij*_ is the coupling factor between molecules *i* and *j*. Since population *x*_*i*_ grows exponentially in the absence of interactions between species, the rate of change of *x*_*i*_ increases at the intrinsic growth rate *a*_*i*_. Each population is limited by its own population at the self-inhibition rate *b*_*i*_; molecules *x*_*i*_ and *x*_*j*_, interact with a coupling strength *c*_*ij*_. In the Eq. 1e, *z* is the myofibril population, and its rate of change is given by:

$$f\left(x_{3},x_{4}\right)=\left\{\begin{array}{ccc} 0 & if & z<z^{\text{min}}\;\;\;or\;\;\;z>z^{\text{Max}}\\ 0 & if & x_{4}<x_{4}^{0}\;\;\;and\;\;\;x_{3}<x_{3}^{0}\\ k_{1}\left(x_{4}-x_{4}^{0}\right)-k_{2}\left(x_{3}-x_{3}^{0}\right) & if & x_{4}>x_{4}^{0}\;\;\;and\;\;\;x_{3}>x_{3}^{0}\\ -k_{2}\left(x_{3}-x_{3}^{0}\right) & if & x_{4}<x_{4}^{0}\;\;\;and\;\;\;x_{3}>x_{3}^{0}\\ k_{1}\left(x_{4}-x_{4}^{0}\right) & if & x_{4}>x_{4}^{0}\;\;\;and\;\;\;x_{3}<x_{3}^{0}, \end{array}\right.$$ (2)

which depends on 1) the difference between populations *x*_3_ and *x*_4_, and their respective thresholds $x_{3}^{0}$ and $x_{4}^{0}$ , 2) the minimum and maximum myofibril populations *z* ^min^ and *z* ^Max^, and 3) *k*_1_ and *k*_2_ as parameters for protein synthesis and degradation, respectively. In the function given in Eq. 2, the first line indicates the rate of change of the myofibril population must be zero if either the minimum or maximum size is reached; the second line indicates that if populations *x*_4_ and *x*_3_ are both below their thresholds, the rate of change of the myofibril population is also zero; the third line indicates that a balance between atrophy and hypertrophy occurs when *x*_4_ and *x*_3_ are both above their thresholds; the fourth line indicates that if only *x*_3_ is above its threshold, then pure atrophy occurs; and finally, the fifth line indicates that if only *x*_4_ is above its threshold, then pure hypertrophy occurs.

The main outcome of the dynamical model (Eqs 1a, 1b, 1c, 1d, 1e) is the rate of change of population *z*, i.e., $f\left(x_{3},x_{4}\right)$ ; this function will be used as the input of the growth tensor. We address this procedure in Supplementary Appendix SA.1.

#### 2.1.2 Mechanical model

The highly organized arrangement of muscle fibers explains the transversely isotropic mechanical behavior of skeletal muscle tissue (Van Loocke et al., 2008; Morrow et al., 2010; Takaza et al., 2013); therefore, the mechanical response is described using a hyperelastic, transversely isotropic constitutive model (Holzapfel, 2000). In this work we used a multiplicative decomposition approach of the deformation gradient ***F*** (Holzapfel, 2000); the decomposition splits the deformation gradient into volume-changing (volumetric) and volume-preserving (isochoric) parts; then, we used an additional multiplicative decomposition of the isochoric part during contraction (Grasa et al., 2012).

The first decomposition is $F=F_{\text{vol}}\;\bar{F}$ , where the volumetric part of the deformation gradient is ***F***_vol_ = *J* ^1/3^ ***I***, and the isochoric part is $\bar{F}=J^{-1/3}\;F$ (also known as modified deformation gradient), *J* is the determinant of ***F*** (also known as the volume fraction), and ***I*** is the second order identity tensor. The additional decomposition during contraction splits the modified deformation gradient, $\bar{F}={\bar{F}}_{e}\;{\bar{F}}_{a}$ , into an elastic component ${\bar{F}}_{e}$ required for compatibility, and an active component ${\bar{F}}_{a}$ to describe active deformation due to contraction (Grasa et al., 2012):

$${\bar{F}}_{a}=\lambda_{a}\;m{}^{\circ}⊗m{}^{\circ}+\lambda_{a}^{-1/2}\left(I-m{}^{\circ}⊗m{}^{\circ}\right),$$ (3)

where *λ*_*a*_ characterizes the active stretch of muscle fibers during contraction, and **m**° is the direction of the muscle fibers.

The use of the multiplicative decomposition leads to consider three contributions to the strain energy function: a volumetric contribution to ensure the nearly incompressible material behavior of the tissue, a passive contribution to account for elastic deformations, and an active contribution to describe muscle contraction. Within this paper, we assume the following strain energy function (SEF):

$$\Psi =\overset{\text{Volumetric}}{\overset{︷}{\Psi_{\text{vol}}\left(J\right)}}+\overset{\text{Passive}}{\overset{︷}{\Psi_{p}\left({\bar{I}}_{1},{\bar{I}}_{4}\right)}}+\overset{\text{Active}}{\overset{︷}{\Psi_{a}\left({\bar{J}}_{4},\lambda_{a},\beta \right)}},$$ (4)

where *J* and *λ*_*a*_ were defined previously; ${\bar{I}}_{1}$ is the first invariant of the modified Cauchy-Green tensor $\bar{C}={\bar{F}}^{T}\;\bar{F}$ ; ${\bar{I}}_{4}$ characterizes the stretch of the collagen fibers, ${\bar{I}}_{4}=a{}^{\circ}\cdot \bar{C}\;a{}^{\circ}$ , where **a**° is the direction of the collagen fibers; ${\bar{J}}_{4}$ characterizes the elastic stretch of the muscle fibers during contraction, ${\bar{J}}_{4}=m{}^{\circ}\cdot \bar{C_{e}}\;m{}^{\circ}$ , where **m**° was defined previously, ${\bar{C}}_{e}={\bar{F}}_{a}^{-T}\;\bar{C}\;{\bar{F}}_{a}^{-1}$ , and ${\bar{F}}_{a}$ was defined in Eq. 3; and *β* is the activation level of the muscle during contraction;

Following Martins et al. (1998), the volumetric contribution is assumed to be

$$\Psi_{\text{vol}}\left(J\right)=\frac{1}{D}{\left(J-1\right)}^{2},$$ (5)

where *D* is a property of the material that controls the bulk modulus.

The passive contribution (Grasa et al., 2011) is:

$$\begin{array}{c} \Psi_{p}\left({\bar{I}}_{1},{\bar{I}}_{4}\right)=\;c_{1}\left({\bar{I}}_{1}-3\right)+\Psi_{f}\left({\bar{I}}_{4}\right), \end{array}$$ (6)

with

$$\Psi_{f}\left({\bar{I}}_{4}\right)=\left\{\begin{array}{ccc} 0 & if & {\bar{I}}_{4}<{\bar{I}}_{40}\\ \frac{c_{3}}{c_{4}}\left[\exp\left(c_{4}\left({\bar{I}}_{4}-{\bar{I}}_{40}\right)\right)-c_{4}\left({\bar{I}}_{4}-{\bar{I}}_{40}\right)-1\right] & if & {\bar{I}}_{4}>{\bar{I}}_{40}, \end{array}\right.$$ (7)

where ${\bar{I}}_{40}$ accounts for the uncurled length of the collagen fibers when these fibers start to stress; and *c*_1_, *c*_3_ and *c*_4_ are material parameters.

**FIGURE 2:**
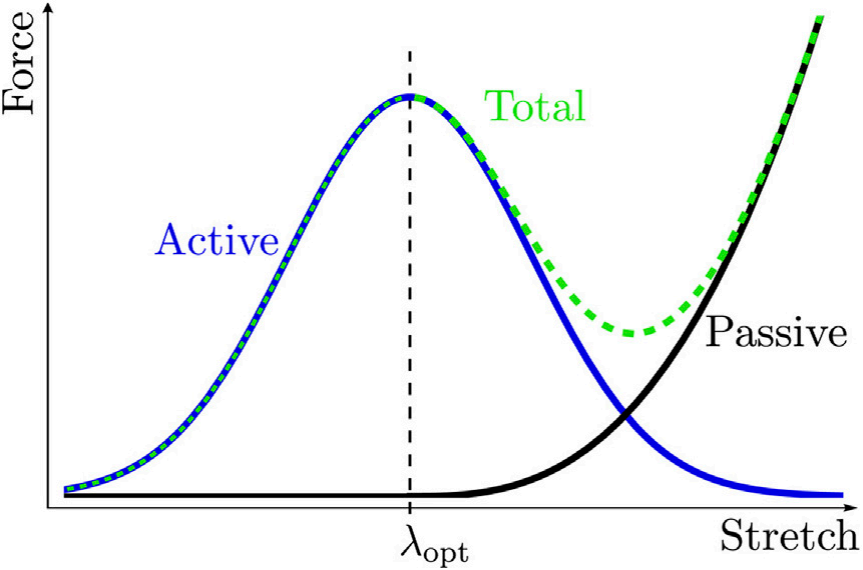
Force-Stretch relation for a muscle. *λ*_opt_ is the stretch where the muscle exerts its maximum force, decreasing or increasing stretch will produce a drop in the generated force. During active response, the maximum isometric tension *σ*_*o*_ characterizes the maximum force, and the active stretch *λ*_a_ characterizes stretch; whereas during passive response, the stretch of collagen fibers ${\bar{I}}_{4}$ characterizes stretch.

Finally, the active contribution (Ramírez et al., 2010; Grasa et al., 2012) is:

$$\Psi_{a}\left({\bar{J}}_{4},\lambda_{a},{\dot{\lambda }}_{a},\beta \right)=\sigma_{o}\;f_{1}\left(\lambda_{a}\right)f_{2}\left({\dot{\lambda }}_{a}\right)f_{3}\left({\bar{J}}_{4}\right)\beta \left(t\right),$$ (8)

where ${\bar{J}}_{4}$ and *λ*_*a*_ were defined previously, ${\dot{\lambda }}_{a}$ is the active stretch rate of the muscle fibers during contraction, and *β* is the activation function; *σ*_*o*_ is the maximum isometric tension that characterizes the maximum force that the organ can exert; function *f*_1_ is the force–stretch relation that represents the active behavior in Figure 2; function *f*_2_ is the force-velocity relation that considers how the maximum force generated depends on how fast a muscle contracts; function *f*_3_ is the energy related to elastic deformation of cross bridges and titin, (Stålhand et al., 2011; Hernández-Gascón et al., 2013); and the activation function *β* controls the force generated by the muscle at time *t* (full activation produces maximum force, zero activation produces a force equal to zero).

The adaptation process takes place in a time course much longer than the activation time course; therefore, the force-velocity relation is not considered relevant in our model. Following Grasa et al. (2012), the functions in Eq. 8 are defined as follows:

$$\begin{array}{cc} f_{1}\left(\lambda_{a}\right) & =\exp\left[-\frac{1}{2}{\left(\frac{\lambda_{a}-\lambda_{\text{opt}}}{1-\alpha }\right)}^{2}\right]\\ f_{2}\left({\dot{\lambda }}_{a}\right) & =1\\ f_{3}\left({\bar{J}}_{4}\right) & =\frac{2}{3}\left(2\;{\bar{J}}_{4}^{3/2}-\frac{3}{2}\;{\bar{J}}_{4}-\frac{1}{2}\right)\\ \beta \left(t\right) & :\text{proportional to the number of activation steps,} \end{array}$$ (9)

where *λ*_opt_ characterizes the optimal length where muscle fibers produce their maximum force, and *α* is a parameter that characterizes how fast the force decays around *λ*_opt_.

The mechanical model allows the description of the deformations of muscle tissue. When we consider a change in size due to muscle hypertrophy or atrophy, it will affect the deformation gradient via the growth tensor that is described in the next section.

### 2.2 Mechanobiological model

In the previous sections, the biochemical and mechanical models that compose our multi-scale mechanobiological model were described. In this section we introduce the concepts to interrelate those independent models.

To describe the coupling procedures between the independent models, we need two conceptual stages and two coupling functions. First, we define the two stages as follows: a *training session* that typically ranges from minutes to hours, occurs when external loading conditions stimulate the biochemical pathway, and allows the assessment of CSA and force according to the mechanical model; and a *growth period* that typically ranges from days to weeks, occurs after each training session, and promotes the evolution of the myofibril population *z* according to the biochemical model. The intention of the two functions, *growth multiplier* and *force-activation relation*, is to build a mathematical connection between the different scales of the independent models and will be briefly described in the paragraphs below. Details of the construction of the functions and the coupling procedures are presented in Supplementary Appendices SA, SB.

The growth of the muscle structure is characterized by the growth tensor ***F***_***g***_, which, following a multiplicative decomposition (Lee, 1969; Rodriguez et al., 1994; Stålhand et al., 2008), requires an elastic deformation ***F***_***e***_ to ensure compatible configurations while the muscle grows:

$$F=F_{e}\;F_{g}$$ (10)

The growth tensor ***F***_***g***_ links the rate of change of myofibrils $f\left(x_{3},x_{4}\right)$ to the increase in cross-sectional area (A) of the muscle structure by using a growth multiplier $G\left(t\right)$ defined as:

$$G\left(t\right)=\frac{f\left(x_{3},x_{4}\right)\;\Delta t}{\kappa \;A\left(t-\Delta t\right)}+1,$$ (11)

where *κ* is a proportionality constant in myofibrils/cm^2^, and Δ*t* is the time step of the evolution of the muscle structure during the growth period. Details about the construction of the growth multiplier are presented in Supplementary Appendix SA.1.

In our model, the growth tensor follows the description given by Göktepe et al. (2010), and in analogy to Eq. 3, we have,

$$F_{g}=F_{g}\left(t\right)=m{}^{\circ}⊗m{}^{\circ}+G{\left(t\right)}^{1/2}\left(I-m{}^{\circ}⊗m{}^{\circ}\right),$$ (12)

where $G\left(t\right)$ , defined by Eq. 11, produces an increase in the area transverse to vector ***m*** °. ***m*** ° and ***I*** were defined previously.

The biochemical model is now coupled to the mechanical model by converting $f\left(x_{3},x_{4}\right)$ (which results from the biochemical model in Section 2.1.1) into the growth tensor (according to Eqs 11, 12), and by operating the growth tensor over the current muscle structure. Details of the implementation of this procedure are presented in Supplementary Appendix SA.2.

Now that we linked the biochemical to the mechanical model, we need to close the feedback loop by linking the mechanical to the biochemical model. In this regard, the force-activation relation F(A,β) links the active response of the mechanical model to the rate of change of myofibrils $f\left(x_{3},x_{4}\right)$ by means of the inverse function β(A,F) . The procedures to build both the function F(A,β) and its inverse function β(A,F) are described in Supplementary Appendix SB.1.

The mechanical model is coupled to the biochemical model by modifying the protein synthesis rate (*k*_1_ in Eq. 2). This modification uses the function β(A,F) as feedback; the concepts for implementing this procedure are presented in Supplementary Appendix SB.2.

**FIGURE 3:**
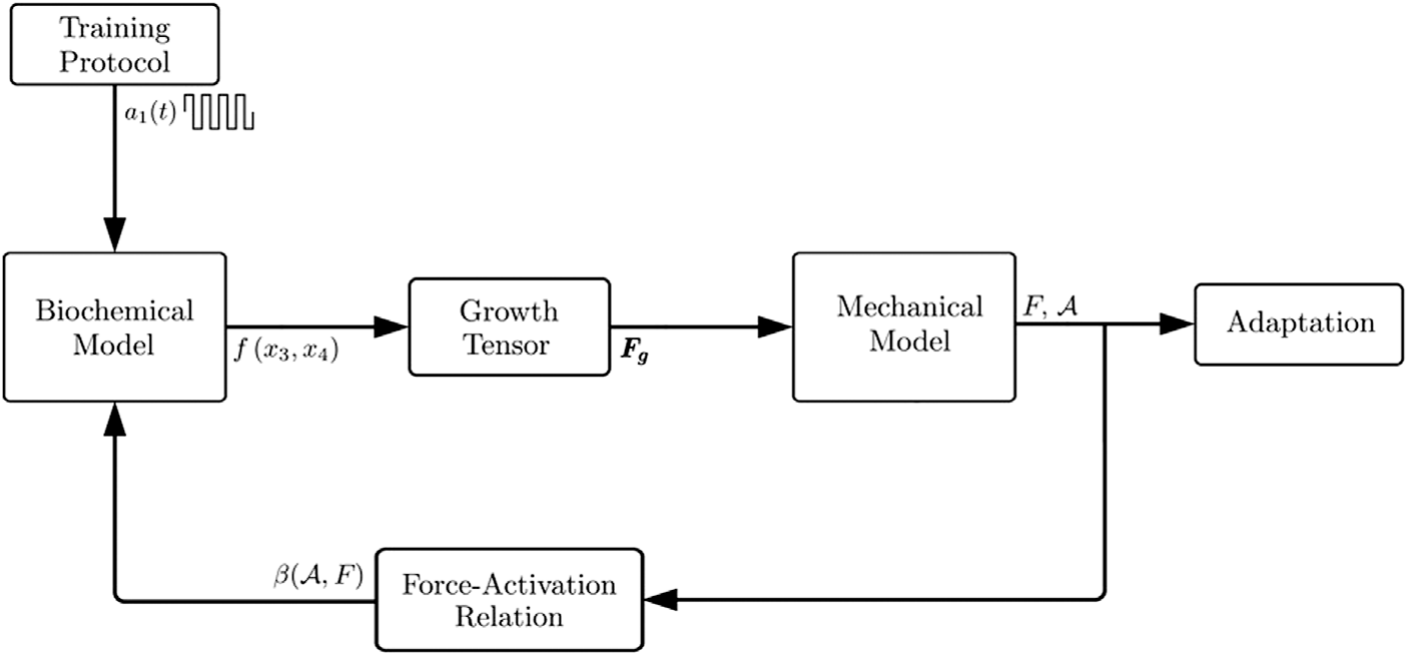
Algorithm for the mechanobiological model for muscle adaptation. Each function outside a block is an output of the closer block and an input for the next block. *f* is the rate of change of the myofibril population, ***F***_***g***_ is the growth tensor, *F* and A are the force and CSA of the updated muscle structure, and β(A,F) is the inverse function of the *force-activation* relation at CSA A .

In summary, the biochemical model is coupled to the mechanical model by means of the growth tensor, and the mechanical model is coupled to the biochemical model by means of the force-activation relation. A full algorithm of the computational model is shown in Figure 3.

### 2.3 Numerical experiments

Table 1 shows the values of the equation parameters and initial conditions required for the implementation of the biochemical model. The training input (*a*_1_(*t*) function) was based on the experimental protocol given in DeFreitas et al. (2011). Subjects in that study performed a total of 24 1-hour sessions on days 1, 3, and 5 of every week. The intensity of training was approximately 80% of the one repetition maximum (1-RM) force. As defined in Section 2.2, the *training session* is the 1-hour training session, while the *growth period* is the time right after training. The training protocol was implemented by the following two-state function *a*_1_(*t*) that triggers the biochemical model:

$$a_{1}\left(t\right)=\left\{\begin{array}{cc} \beta \;I\;F_{\text{max}}\;\; & \text{if }\;t\;\epsilon \text{ training session}\\ a_{10}\;\; & \text{if }\;t\;\epsilon \text{ growth period} \end{array}\right.$$ (13)

Where *β* = 2/50 (hours*kgf)^−1^; *I* is the fraction of the maximum force (*F*_max_ in kgf) that the muscle can produce; *a*_10_ is given in Table 1.

**TABLE 1:** Parameters and initial conditions for the biochemical model. For equation system 1: intrinsic growth rates (*a*_*i*_), self-inhibition rates (*b*_*i*_), coupling strengths between species (*c*_*ij*_). For the rate of change of the myofibril population (Eq. 2): Minimum value (*z* ^min^); maximum value (*z* ^Max^); threshold for *x*_3_ $\left(x_{3}^{0}\right)$ ; threshold for *x*_4_ $\left(x_{4}^{0}\right)$ ; protein synthesis rate (*k*_1_); and protein degradation rate (*k*_2_). Initial conditions of equation system 1: for molecules (*x*_*i*_(0)), and for myofibril population (*z*(0)). *a*_10_ and *a*_20_ are reference values for functions *a*_1_(*t*) and *a*_2_(*t*). Values from Villota-Narvaez et al. (2021).

Parameter	Value	Units
*a*_10_	9.000 × 10^–2^	hours^−1^
*a*_20_	4.875 × 10^–1^	hours^−1^
*a*_3_	1.068 × 10^–2^	hours^−1^
*a*_4_	4.635 × 10^–3^	hours^−1^
*b*_1_	2.000 × 10^0^	hours^−1^ (a.u.)^−1^
*b*_2_	5.000 × 10^–1^	hours^−1^ (a.u.)^−1^
*b*_3_	2.000 × 10^–2^	hours^−1^ (a.u.)^−1^
*b*_4_	1.000 × 10^–2^	hours^−1^ (a.u.)^−1^
*c*_21_	2.846 × 10^–1^	hours^−1^ (a.u.)^−1^
*c*_32_	2.000 × 10^–3^	hours^−1^ (a.u.)^−1^
*c*_42_	1.139 × 10^–3^	hours^−1^ (a.u.)^−1^
*c*_43_	2.500 × 10^–3^	hours^−1^ (a.u.)^−1^
*z* ^min^	0.5000	a.u.
*z* ^Max^	1.300	a.u.
$x_{3}^{0}$	4.340 × 10^–1^	a.u.
$x_{4}^{0}$	4.690 × 10^–1^	a.u.
*k*_1_	2.500 × 10^–2^	hour^−1^
*k*_2_	1.900 × 10^–2^	hour^−1^
*x*_1_(0)	1.000 × 10^–2^	a.u.
*x*_2_(0)	9.880 × 10^–1^	a.u.
*x*_3_(0)	4.318 × 10^–1^	a.u.
*x*_4_(0)	4.692 × 10^–1^	a.u.
*z*(0)	1.000 × 10^0^	a.u.

*a*_2_(*t*), required for the dynamics of *x*_2_, was defined as:

$$\frac{da_{2}}{dt}=\frac{1}{2}\left(-\frac{1}{\tau_{h}}-\frac{t-t_{1}}{\tau_{h}^{2}}\right)\exp\left[\frac{t-t_{1}}{\tau_{h}}\right],\;a_{2}\left(0\right)=a_{20}$$ (14)

where *τ*_*h*_ = 6 h, *t*_1_ = 17 h, *a*_20_ is given in Table 1. In the case when no exercise signal is applied, *a*_2_(*t*) = *a*_20_.

Table 1 shows parameters and initial conditions for the biochemical model, those values were reported in Villota-Narvaez et al. (2021). In that study, homeostatic concentrations of the molecules involved in the pathway, taken from Léger et al. (2006) and Bickel et al. (2005), were normalized to the concentration of IGF1, and those values were used as initial conditions; the myofibril population was set to 1; parameters for the equation system 1 were fitted to experimental evidence on atrophy and used in this article for the hypertrophy case.

Equation system 1a, 1b, 1c, 1d, 1e was solved by means of a Runge-Kutta fourth-order method in a Fortran routine. The time step was *dt* = 0.05 h and the total time simulated was 8 weeks.

The difference in muscle size from *t* to *t* + *dt* is negligible; therefore, we use a much longer time step Δ*t* = 1 h for the adaptation in size during the growth period. By solving equation system 1a, 1b, 1c, 1d, 1e, we have 1/dt data points for each simulated hour and for each biochemical variable. From all those data, we used the values of *f*(*x*_3_, *x*_4_) every Δ*t*/*dt* steps to build the growth tensor.

**FIGURE 4:**
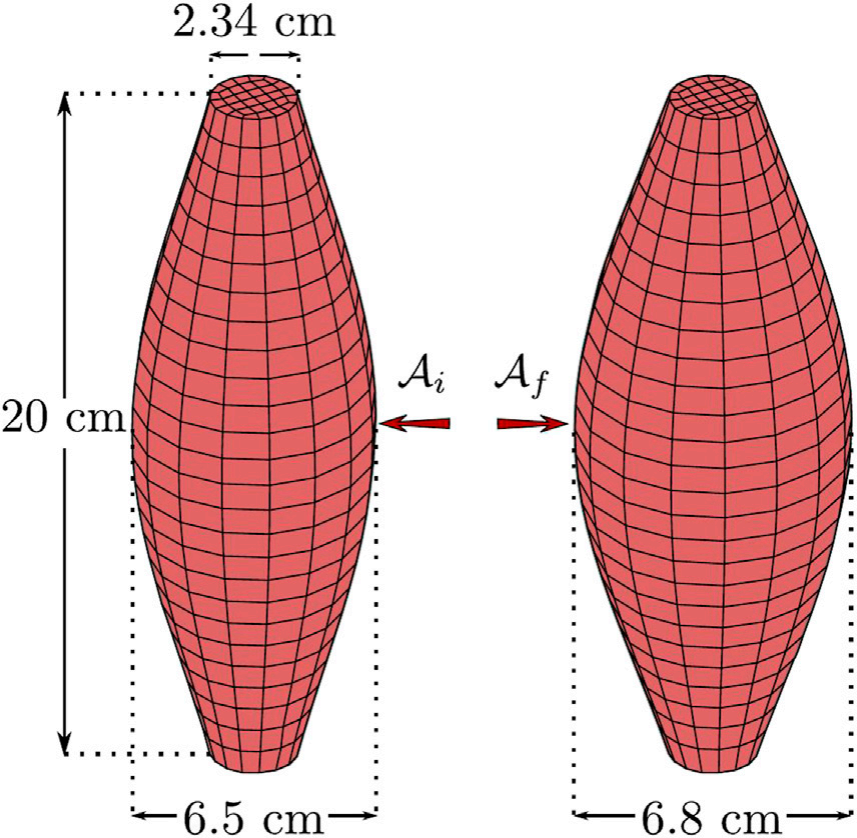
Comparison between muscle structure before and after the training protocol.

Our FEM procedure uses an idealized muscle structure as shown in Figure 4. The initial structure was built from a cylindrical shape of 20 cm long, 2.34 cm diameter on the top and bottom, and 6.5 cm diameter half height. The geometry was discretized into a structured mesh of 832 8-noded brick elements with a total of 1107 nodes (see Figure 4). As boundary conditions, nodes at top and bottom surfaces were kept fixed for displacement and rotation. Table 2 shows the set of parameters for the implementation of the mechanical model.

**TABLE 2:** Parameters for the mechanical model.

Parameter	Value	Units	References
1/*D*	100.0	MPa	Grasa et al. (2011), Grasa et al. (2012)
*c*_1_	0.0100	MPa	Calvo et al. (2010)
*c*_3_	0.0543	MPa	Calvo et al. (2010)
*c*_4_	6.86	—	Calvo et al. (2010)
${\bar{I}}_{40}$	1.2544	—	Calvo et al. (2010)
*σ*_*o*_	0.100	MPa	Miller et al. (2015)
*λ*_opt_	1.000	—	Ramírez et al. (2010)
*α*	0.83616	—	Ramírez et al. (2010)

The implementation of the mechanical model depends on the stage of the evolution. First, the implementation during the growth period assumes the growth multiplier given in Eq. 11, the growth tensor given by Eq. 12, the activation parameter *β* = 0, and Δ*t* = 1 h. Second, during the training session, the growth tensor is equal to the identity tensor; the activation parameter increases from 0 to 1 and then decreases back to 0; and Δ*t* only counts the activation steps (it does not have a physical meaning during the evaluation of force). The mechanical response of the tissue was simulated by means of a UEL subroutine, implemented in Fortran, linked to the solving procedures in the specialized software ABAQUS 3DEXPERIENCE R2017x (Dassault Systemes USA, Waltham, MA).

We will compare the CSA evolution from our mechanobiological model to the adaptation results obtained by DeFreitas et al. (2011). In their study, the CSA of the right thigh muscle was measured every week by using a peripheral quantitative computed tomography.

The applicability of our model is explored by simulating the hypertrophy results under different training frequencies, different values of the proportionality constant *κ* defined in Eq. 11, and variations of the modified protein synthesis rate.

For an estimation of the effect of parameter values of Eqs 1a, 1b, 1c, 1d, 1e, we tested variations of the parameters within 1% of the value presented in Table 1; we calculated the Root Mean Squared Error RMSE of the CSA relative to the hypertrophy results reported by DeFreitas et al. (2011), and the maximum increase in CSA. We varied one parameter while all the others remained fixed.

## 3 Results

The mechanobiological model in Section 2.2 was tested under the numerical experiments described in Section 2.3. The main result of the numerical experiments was the increase in CSA of the muscle structure at the end of three different stages of the algorithm (see Figure 3). Relative to the experimental results, the CSA evolution improves after each stage: first, at the end of the biochemical model (myofibril population *z*); second, at the end of the mechanical model before the implementation of the feedback from the mechanical to the biochemical model ( A that results by the use of *f*(*x*_3_, *x*_4_) in the growth tensor); and third, at the end of the mechanical model after the feedback was included ( A that results by the use of β(A,F) in *f*(*x*_3_, *x*_4_), and *f*(*x*_3_, *x*_4_) in the growth tensor).

**FIGURE 5:**
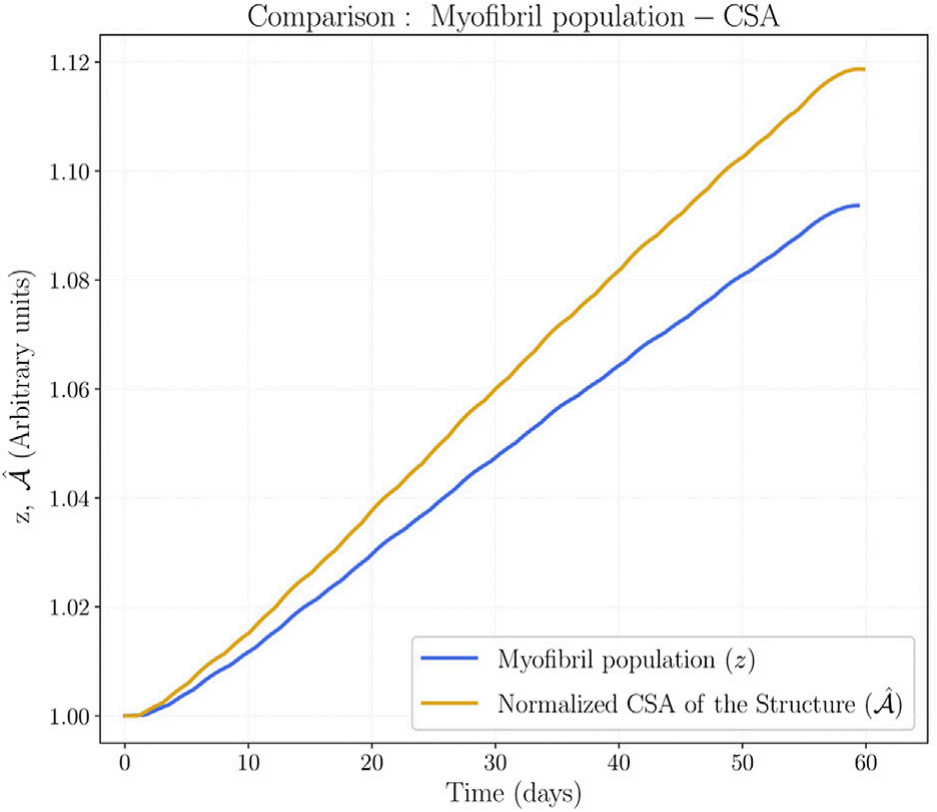
Cross Sectional Area and myofibril population comparison. These results were obtained by considering full activation (*β* = 1) for the whole training protocol, and no feedback from the mechanical to the biochemical model.

The first two stages show that the CSA of the muscle structure differs from the myofibril population of the biochemical prediction, as seen in Figure 5. This difference is explained by the elastic response of the material that enforces compatible configurations. In this regard, since the mechanical properties of the muscle tissue do not change due to adaptation processes, the elastic deformation contribution of the deformation gradient (Eq. 10) does not depend on our biochemical parameters. Hence, the difference between CSA and biochemical prediction shown in Figure 5 cannot be avoided because the biochemical model only controls the growth tensor contribution of the deformation gradient.

**FIGURE 6:**
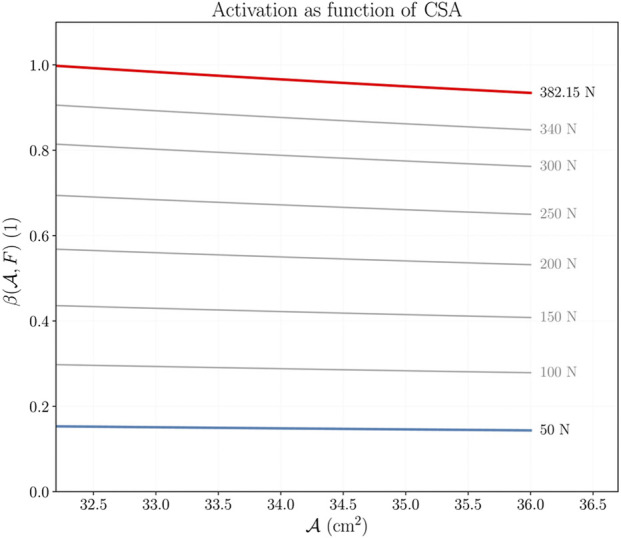
Activation level *β* as a function of the CSA A for different force levels. The activation required to produce a fixed force decreases as the CSA of the structure increases. Each curve results by fixing the value of *F* and calculating *β* according to Supplementary Equations S24, S25.

Before the results of the third stage, we need to consider function β(A,F) (which is the inverse function of the force-activation relation and link from the mechanical to the biochemical model). Function β(A,F) (Figure 6), obtained following the procedure described in Supplementary Appendix SB.1, shows that a larger muscle requires a smaller activation to produce a fixed force.

Assuming an increasing CSA, the following observations about function β(A,F) justify the necessity to feedback the biochemical model: first, the decrease in the activation required to produce a fixed force implies that the muscle tissue receives a decreasing intensity of stimulus if the training load is fixed during the training protocol; second, the variation on the activation required with lighter loads is almost negligible and supports the recommendation of training with intermediate to high loads; and third, the variation on the activation required with heavier loads is noticeable and supports the recommendation of training with increasing loads. These observations can be related to early neuronal adaptation, which can occur before significant hypertrophy happens. However, since our model ignores the neuronal variable, further analysis is necessary.

Now, let us consider the CSA after stage three, which includes the function β(A,F) as feedback. In the biochemical model, the size of the myofibril population *z* is strongly dominated by the size of population *x*_4_. However, we assumed that population *x*_4_ should remain close to its threshold $x_{4}^{0}$ to have steady-state solutions; therefore, we included the feedback directly into the protein synthesis rate *k*_1_ (Eq. 2) rather than in the equation for the rate of change of *x*_4_. The modified protein synthesis rate *k*_1_ is given by:

$$k_{1}=k_{10}*\left(d_{1}\;\beta \left(A,F\right)-d_{2}\right)$$ (15)

where *k*_10_ is the *k*_1_ value used in the biochemical system without feedback, and dimensionless parameters *d*_1_ and *d*_2_ allow us to adjust the strength of the coupling; in our simulations: *d*_1_ = 20.40, and *d*_2_ = 18.907.

**FIGURE 7:**
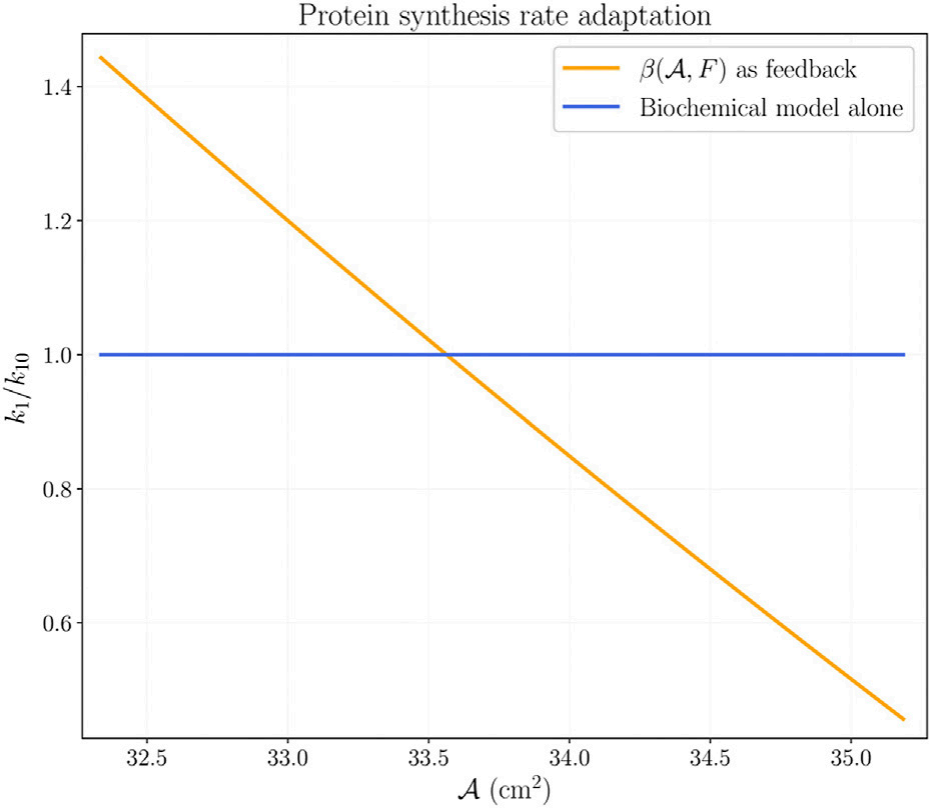
Protein synthesis rate *k*_1_ given by Eq. 15, at parameters *d*_1_ = 20.40 and *d*_2_ = 18.907. Here, we compare the growth rate *k*_1_ of the original biochemical system Eqs 1a, 1b, 1c, 1d, 1e with the modified value of *k*_1_ using the feedback from the mechanical response.

**FIGURE 8:**
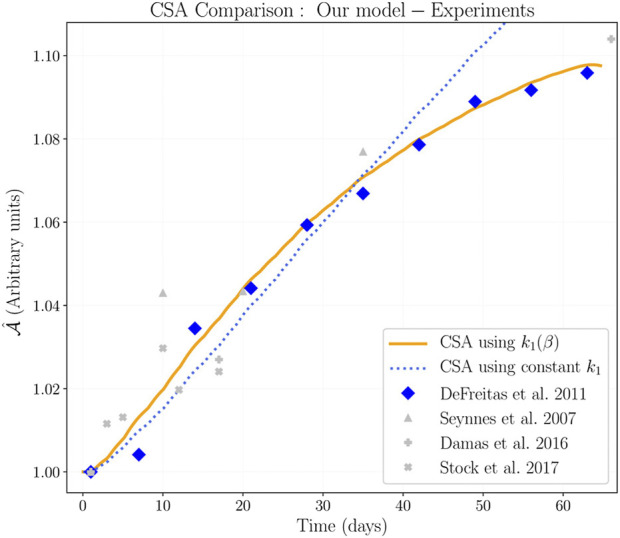
Normalized CSA adaptation due to training. Experimental results compared to our mechanobiological model. The activation function shown in Figure 6 was used to feedback equation system Eqs 1a, 1b, 1c, 1d, 1e. The modified protein synthesis rate *k*_1_(*β*) was defined in Eq. 15, and replaces the constant value of the protein synthesis rate in function $f\left(x_{3},x_{4}\right)$ defined through Eq. 2. We simulated the training protocol of DeFreitas et al. (2011), and fitted parameters *d*_1_ and *d*_2_ to minimize the RMSE.

**FIGURE 9:**
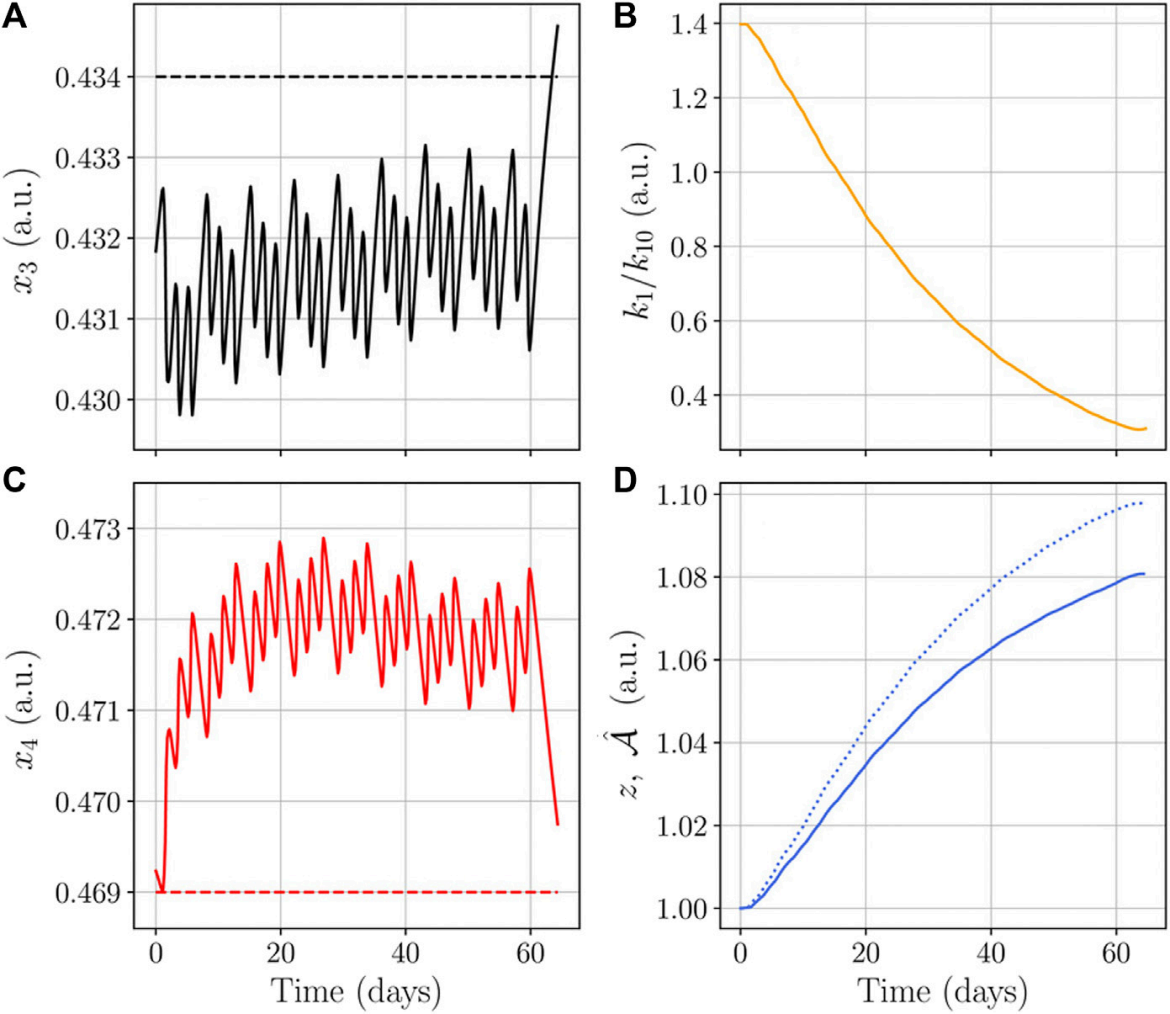
Biochemical variables and protein synthesis rate during the CSA adaptation shown in Figure 8. Dashed lines represent threshold levels in figures **(A,C)**; these figures show that periodic training leads to oscillations of *x*_3_ below its threshold, and *x*_4_ above its threshold; those levels favor hypertrophy according to Eq. 2. Figures **(B,D)** show that protein synthesis rate decreases as *z* increases. And figure **(D)** shows the results presented in Figure 5 after the use of function β(A,F) as feedback; recall that the elastic response required for compatible configuration explains the difference between *z* (continuous line) and $\hat{A}$ (dotted line). (a.u., arbitrary units).

Figure 7 shows a comparison of the ratio *k*_1_/*k*_10_ of the biochemical system alone, and the same ratio using the coupling relation β(A,F) as feedback. At the initial CSA, the coupling relation produces a greater value of *k*_1_ than the value of *k*_10_. A greater value of *k*_1_ produces a faster growth rate during the first weeks of training in agreement with experimental results (Figure 8). The parameter *k*_1_ decreases below *k*_10_ with increasing CSA; this decreasing value of *k*_1_ implies a slower growth rate. Figure 9 shows the time courses of *x*_3_, *x*_4_, *z*, and *k*_1_/*k*_10_ that lead to the CSA shown in Figure 8.

Figure 8 shows our model results when the feedback is used in *k*_1_, and also a comparison with experiments. Our results show that muscle grows faster during the early days of the training period, and the growing speed decreases with time even when training is continued. This means that the protein synthesis rate decreases as the protein content increases until eventually a maximum muscle size is reached. In our model, the protein synthesis rate was initially set constant (before the feedback implementation); when we fed back the biochemical system directly in the protein synthesis rate (according to Eq. 15) our model matches in size and shape the experimental results. Therefore, we argue that the muscle adaptation feedback affects directly the protein synthesis level. In addition, our model shows that a muscle cannot grow indefinitely when driven only by exercise.

**FIGURE 10:**
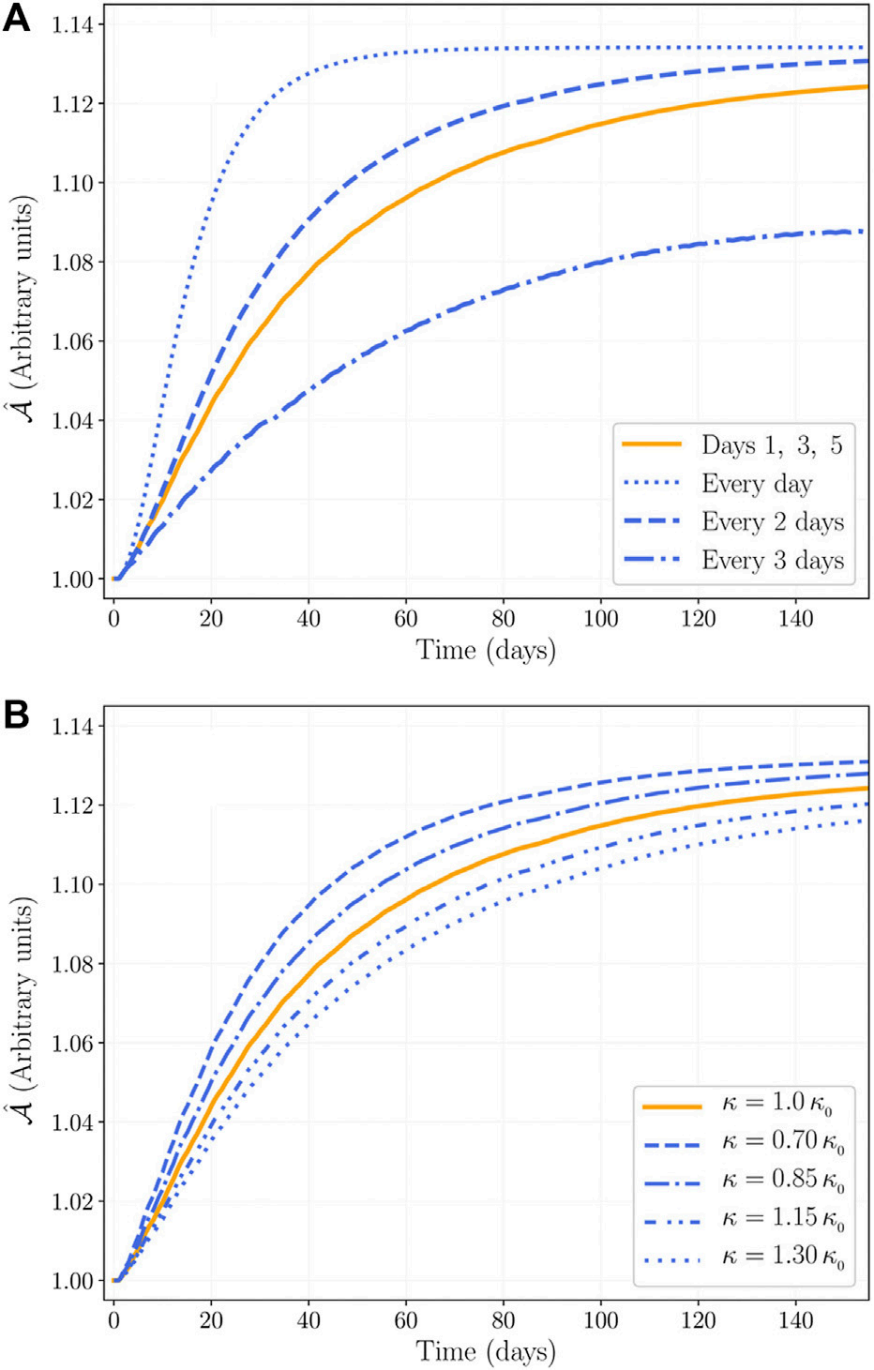
CSA increase under variation of: **(A)** training frequency, and **(B)** the proportionality constant *κ* defined in Supplementary Equation S16, with $\kappa_{0}=1/A\left(0\right)$ .

The evolution of *x*_3_ and *x*_4_ presented in Figures 9A,C is specific for the training protocol of DeFreitas et al. (2011) that consisted in three training sessions per week. Different training frequencies affect the oscillations of *x*_3_ and *x*_4_, whose values lead to different responses (according to Eq. 2) depending on how frequently *x*_3_ and *x*_4_ cross their thresholds. Figure 10A shows the results of the simulation under different protocols: training every day, every 2 days, every 3 days, and on days 1, 3, and 5 of every week (protocol used by DeFreitas et al. (2011)). Our results show that the maximum CSA increase is affected by training frequency: the more trainings per week, the higher the CSA increase. Our results also suggest that training three times per week slowly leads to a maximum CSA close to the results of training every 2 days, whereas training every 3 days leads to a significantly less CSA increase.

Supplementary Equation S16 allows us to have κ=z(0)/A(0) . In our analysis, a variation of *κ* allows us to compare subjects with different initial myofibril populations; however, as there is no need to consider the number *z*(0), we continue using *z*(0) = 1 as in Table 1, and by using different values of *κ* we are using a relative number of myofibrils. For instance, all the results presented previously were obtained by using κ=1/A(0) (let us call it *κ*_0_); then, the use of *κ* = 0.7 *κ*_0_ represents a subject whose myofibril population is only 70% relative to the reference subject. Figure 10B presents the results of the simulation using different values of *κ*, and the training protocol of DeFreitas et al. (2011). Our results show that subjects with initially larger number of myofibrils reach smaller CSA.

**FIGURE 11:**
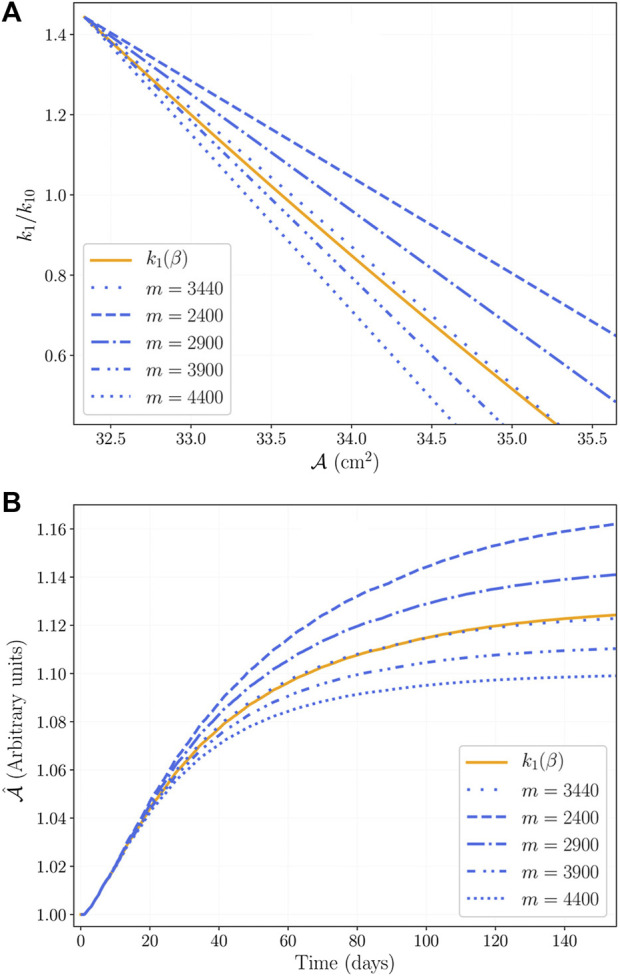
**(A)** is the linear approximation of the *k*_1_/*k*_10_ numerical solution of Figure 7, the variation of the slope represents different protein synthesis responses. **(B)** Evolution of the CSA for the different variations presented in **(A)**.

To evaluate variations of the modified protein synthesis rate in a simplified way, note that the results shown in Figure 7 suggest a straight line approximation to the numerical relation *k*_1_/*k*_10_. We tested such approximation as $k_{1}/k_{10}=b-mA$ , where *m* > 0 is the slope of the line, and *b* is such that *k*_1_/*k*_10_ evaluated at A(0) is a fixed value. Figure 11A shows the protein synthesis rate using different slopes *m*, and includes the numerical result of Figure 7 labeled as *k*_1_(*β*) (which is *k*_1_/*k*_10_ from Eq. 15). Figure 11B presents the results of the simulation using the approximations shown in Figure 11A. First, note that the straight line approximation with *m* = 3440 agrees with the numerical results using *k*_1_(*β*); second, note that all variations produce the same increase in CSA during the first 20 days of training; and third, note that smaller values of *m* lead to larger increases in CSA.

Parameters *a*_10_, *b*_1_, *c*_21_, and *c*_32_ have very little effect (RMSE changes less than 1%, and CSA changes less than 0.2%). Parameters *a*_20_, *b*_2_, *c*_42_, and *c*_43_ have a small effect (RMSE increases up to 3 times the best fit value, while CSA changes less than 2%). Parameters *a*_3_ and *b*_3_ have a considerable effect (RMSE increases up to 8 times the best fit value, while CSA changes about 4%). Parameters *a*_4_ and *b*_4_ have a big effect (RMSE increases up to 16 times the best fit value, while CSA changes up to 9%). None of the tested variations produced saturation in hypertrophy, but zero hypertrophy resulted by 1% smaller value of *a*_4_, and also by 1% larger value of *b*_4_. These results are consistent with the importance of *x*_3_ and *x*_4_, because these variables control the rate of change of *z* (Eq. 1e).

## 4 Discussion

Regarding results shown in Figure 8. Experiments show that muscle grows even in the early days of a training period. According to Seynnes et al. (2007) and DeFreitas et al. (2011), the early increase in CSA can be considered as hypertrophy; but Damas et al. (2016) argue that a major contribution could be related to edema. To solve the edema observation, Stock et al. (2017) measured the CSA increase under a concentric-only training; they showed that hypertrophy is small but detectable during the first training sessions. We can see that the increase in CSA is similar in all cases, even when the training protocol avoids edema. Thus, we can argue that, although our model ignores muscle damage and considers isometric contraction, our results are in good agreement to regular and concentric-only training.

We found that training frequency has a big impact in CSA increase assuming a constant volume per training session. However, some authors suggest that training volume per week is a variable more important than frequency Figueiredo et al. (2018), Schoenfeld et al. (2017). Evidence shows that training once per week leads to the same hypertrophy results than training 2 or 3 times per week when training is volume equated Brigatto et al. (2019), Gentil et al. (2015), Grgic et al. (2019). Our model requires more details in the training signal to address training volume and other features of different training protocols.

Regarding the effect of *κ*, myofibril size increases during skeletal muscle growth, and, after reaching a threshold size, they split (Goldspink, 1970; Jorgenson et al., 2020). This evidence may explain our results in Figure 10B: the more myofibrils per unit area, the smaller their size; therefore, larger myofibrils increase the myofibril population sooner than smaller ones, because larger myofibrils split easier than smaller ones. In this sense, smaller values of *κ* (larger initial myofibril sizes) lead to larger hypertrophy results.

Our results on protein synthesis rate adaptation are consistent with evidence on training status. According to Phillips et al. (2002), untrained subjects show a higher protein synthesis than trained subjects. In our model, training status relates to the force-activation relation β(A,F) , because we propose that this function modifies the protein synthesis rate according to Eq. 15. Our model lacks of a time description of protein synthesis rate, (time course of protein synthesis was reviewed by Damas et al. (2015), but we argue that CSA and force adaptation are the variables that define hypertrophy results in the long term. One last comment on the protein synthesis rate, the straight line approximation only depends on CSA, and although our numerical result is a function that depends on CSA and force, such approximation can be interpreted as a way to test therapeutic treatment with the aim of increasing maximum CSA, or accelerate hypertrophy results.

We proposed a multi-scale mechanobiological model for muscle adaptation. Starting at the biochemical base of the IGF1-AKT signaling pathway to predict how the protein content inside a muscle fiber evolves, we defined a growth multiplier and subsequently a growth tensor that allowed us to connect the cellular scale of the adaptation to the organ scale by means of the mechanics of growing tissue. Furthermore, the characteristic adaptation in force allowed us to build a function that describes how the activation of a muscle changes during the adaptation process. We proposed that this function affects the protein synthesis rate, and in this way the function connects the organ scale to the cellular scale.

Our multi-scale mechanobiological model is triggered by an exercise training protocol, and allows to predict how the protein content of the muscle evolves. We found that the activation required to produce force changes during the training protocol, and we argue that this activation change can be considered as a quantification of common training recommendations regarding intensity and loading increase.

Although many important aspects of the muscle function, such as neural signaling, fatigue, and fiber differentiation were not taken into account, our results show that the methodology of the growth tensor and the feedback function, which are key in our mechanobiological model, is capable of producing remarkable agreement with experiments. Future work will address the inclusion of fiber differentiation and fatigability.

## Data Availability

The original contributions presented in the study are included in the article/Supplementary Material, further inquiries can be directed to the corresponding author.
