# Supplementary material for: Multi-scale mechanobiological model for skeletal muscle hypertrophy
Source: Front Physiol. 2022 Oct 6;13:899784. doi: 10.3389/fphys.2022.899784 (PMC9582841; doi:10.3389/fphys.2022.899784)
Supplement: Supplementary file 1 [file Presentation1.pdf]

Supplementary Material

Appendices

A COUPLING THE BIOCHEMICAL TO THE MECHANICAL MODEL

Muscle structure at the organ scale adapts according to signals at the biochemical scale. First we present the construction the growth multiplier that couples scales, and then we present the procedure to update the muscle structure.

A.1 Constructing the growth multiplier

In sections 2.1.1 and 2.1.2, we presented the mathematical description of the IGF1-AKT signaling pathway and the mechanical response of muscle as a continuous material; however, those descriptions are independent of one another. Now we introduce the growth multiplier, which links the rate of change of myofibrils $f\,(x_3, x_4)$ to the increase in CSA of the continuous material.

If myofibrils are added in parallel, the CSA of a muscle fiber increases; therefore, we assume that the myofibril population z is proportional to the CSA of muscle ($\mathcal{A}$):

$$z\,(t) = \kappa\,\mathcal{A}\,(t) \tag{S16}$$

Where κ is the proportionality constant in myofibrils/cm^2.

As muscle tissue is assumed as a nearly incompressible material, the mass density of the tissue will be considered constant. From a mechanical perspective, the CSA at time t is equal to the growth multiplier at time t acting on the CSA at time $t - \Delta t$

$$\mathcal{A}\,(t) = G\,(t)\;\mathcal{A}\,(t - \Delta t) \tag{S17}$$

From the biochemical perspective, the myofibril population is the outcome of the biochemical system (eq. 1a, 1b, 1c, 1d, 1e)

$$\frac{d\,z}{dt} = f\,(x_3, x_4) \tag{S18}$$

A small increment in z is:

$$\Delta\,z = f\,(x_3, x_4)\;\Delta t \tag{S19}$$

We linked the evolution of z to $\mathcal{A}$ by replacing eq. S16 in the definition of a differential of z:

$$\begin{aligned} \Delta\,z &= z\,(t) - z\,(t - \Delta t) \\ &= \kappa\,\mathcal{A}\,(t) - \kappa\,\mathcal{A}\,(t - \Delta t) \end{aligned} \tag{S20}$$

Using the mechanical evolution of $\mathcal{A}$ given by eq. S17:

$$\begin{aligned}\Delta z &= \kappa\, G\,(t)\;\mathcal{A}\,(t-\Delta t) - \kappa\,\mathcal{A}\,(t-\Delta t)\\ &= \kappa\;(G\,(t)-1)\;\mathcal{A}\,(t-\Delta t)\end{aligned} \tag{S21}$$

From this, we get:

$$G\,(t) = \frac{\Delta z}{\kappa\,\mathcal{A}\,(t-\Delta t)} + 1 \tag{S22}$$

and finally, using the biochemical evolution of z given by eq. S19:

$$G\,(t) = \frac{f\,(x_3, x_4)\;\Delta t}{\kappa\,\mathcal{A}\,(t-\Delta t)} + 1 \tag{S23}$$

We consider $G\,(t)$ as the growth multiplier of the muscle transverse to the fibers. This implies that the transverse components of the growth tensor are modulated by this growth multiplier. In equation S23, Δt is the time increment of geometry of the muscle via the mechanical model, which is not necessarily as small as the time increment required to solve the biochemical model.

A.2 Updating the muscle structure with the growth tensor

Our simulation starts at day 0 with a growth tensor equal to the identity tensor. At day 1 the training protocol function $a_1(t)$ stimulates the biochemical model for the first time; such stimulation lasts a training session. After a training session, the populations of molecules in the biochemical model evolve according to the system of equations presented in section 2.1.1.

The implementation of the mechanobiological model continues as follows:

1. Right before a new training session stimulates the system, the last state of the variables of the biochemical model, the whole history of the $f\,(x_3, x_4)$ function, and the known (current) muscle structure are stored.
2. During the growth period, the rate of change of the population of myofibrils ($f\,(x_3, x_4)$ function) at time t is converted into the growth tensor $\boldsymbol{F_g}(t)$ according to equations S23 and 12.
3. According to the kinematics of finite growth, Göktepe et al. (2010), $\boldsymbol{F_g}(t)$ is applied to the current muscle structure in order to generate the updated structure.
4. The time t is incremented; the process goes back to step 2 if a growth period is occurring, or to step 1 if a new training session is going to stimulate the system. In the latter case, the last stored state of the variables of the biochemical model is used as a new set of initial conditions.

B COUPLING THE MECHANICAL TO THE BIOCHEMICAL MODEL

Biochemical parameters adapt according to muscle size and strength. First we present the construction the force-activation relation that couples scales, and then we present the procedure to update the biochemical model.

B.1 Constructing the force-activation relation

The CSA of the structure changes as a result of applying the growth tensor over the current structure during the growth period. We assume that every updated muscle structure can generate a different force.

The structure right **before** a training session is subjected to an activation cycle of the mechanical model (section 2.1.2) to evaluate how the force generation is affected by the adapting structure: the mechanical model runs over different values of the activation parameter (β) and calculates the corresponding force (F); as β increases from 0 to 1, the force F increases. This relation is what we call *force-activation* relation. In what follows, the CSA of the structure stored **before** a training session will be simply referred to as CSA.

To build the link from the mechanical to the biochemical model, we need a function ($\beta = \beta\,(\mathcal{A}, F)$) that, for a given force (F) and a given CSA ($\mathcal{A}$), describes how activation (β) changes. To address this aim, we implemented the following procedure:

- For each CSA, a set of points from the *force-activation* relation is obtained. A quadratic fit for the inverse relation *activation-force* is calculated (see figure S12):

$$\beta\,(F) = a_{\mathcal{A}} + b_{\mathcal{A}}\,F + c_{\mathcal{A}}\,F^2,$$

  where the subindex $\mathcal{A}$ means that the coefficient depends on a fixed value of CSA.
- Using each CSA and its corresponding coefficients, sets $\{\mathcal{A}, a_{\mathcal{A}}\}$, $\{\mathcal{A}, b_{\mathcal{A}}\}$, $\{\mathcal{A}, c_{\mathcal{A}}\}$ are built.
- A quadratic fit for each set is calculated:

$$\begin{aligned} a(\mathcal{A}) &= a_0 + a_1\,\mathcal{A} + a_2\,\mathcal{A}^2 \\ b(\mathcal{A}) &= b_0 + b_1\,\mathcal{A} + b_2\,\mathcal{A}^2 \\ c(\mathcal{A}) &= c_0 + c_1\,\mathcal{A} + c_2\,\mathcal{A}^2 \end{aligned} \tag{S24}$$

- The functions defined in equation S24, allow us to have an explicit activation function:

$$\beta\,(\mathcal{A}, F) = a(\mathcal{A}) + b(\mathcal{A})\,F + c(\mathcal{A})\,F^2, \tag{S25}$$

In summary, from the *force-activation* relation, we built the function $\beta(F)$ by a quadratic fit. The coefficients of the $\beta(F)$ function depend on the CSA and allow us to built functions $a(\mathcal{A})$, $b(\mathcal{A})$ and $c(\mathcal{A})$ also by quadratic fits. The quadratic function for $\beta(F)$ and the quadratic functions $a(\mathcal{A})$, $b(\mathcal{A})$ and $c(\mathcal{A})$ are combined into the full activation function $\beta(\mathcal{A}, F)$ that allows us to calculate the activation β that generates a given value of force F at a given CSA $\mathcal{A}$ of the muscle structure. Figure S13 shows a diagram of the procedure to build the $\beta(\mathcal{A}, F)$ function.

We assume that the adaptation of the activation (β) affects the evolution of the biochemical system. Therefore, the *Activation* function is the link from the mechanical model to the biochemical model that allows us to have a closed loop for the mechanobiological system.

B.2 Updating the rate of protein synthesis with the force-activation relation

We proposed that the link from the biochemical to the mechanical model is the growth tensor ($\boldsymbol{F_g}$). In addition, we mentioned that the relation between *Activation*, CSA, and force ($\beta(\mathcal{A}, F)$) is the link from the mechanical to the biochemical model. However, as we can see in equation system 1a, 1b, 1c, 1d, 1e there are many possibilities to be affected by the adaptation process; for instance, the growth rate of each molecule or the coupling coefficients. Nonetheless, we propose that the major impact of the muscle structure adaptation (link from the mechanical to biochemical model), should be in the rate of change of the myofibril population ($f\,(x_3, x_4)$, eq. 2), specifically in the rate of protein synthesis k_1.

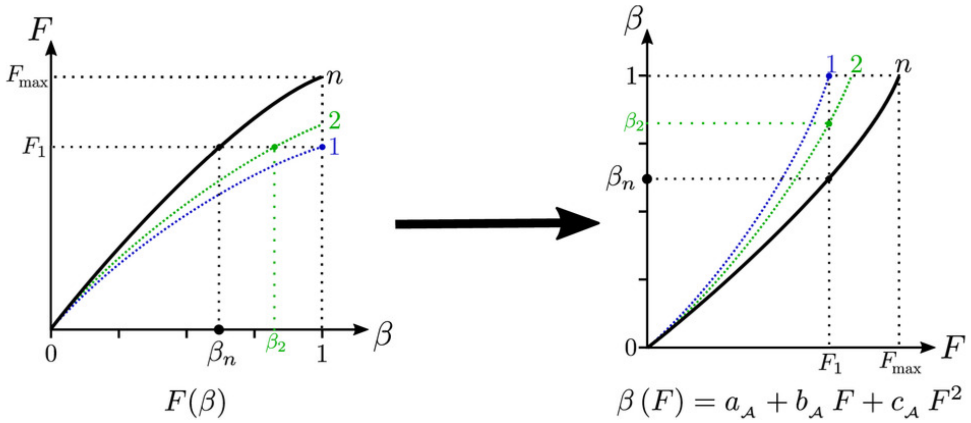

Figure S12. *Force-Activation* relation $F(\beta)$. Force F increases as the activation β increases from 0 to 1. We assume that $F(\beta)$ changes as a result of muscle tissue adaptation (each curve is numbered according to the training session and corresponds to a different CSA). The muscle is able to produce the force F_1 at full activation $\beta = 1$ in the first training session; due to adaptation, the muscle requires a smaller activation β_n to produce the same force after n training sessions. The right sketch shows that, for each different CSA, a curve $\beta(F)$ is fitted by a quadratic function; therefore, there are sets of coefficients: $\{\mathcal{A}, a_{\mathcal{A}}\}$, $\{\mathcal{A}, b_{\mathcal{A}}\}$, $\{\mathcal{A}, c_{\mathcal{A}}\}$.

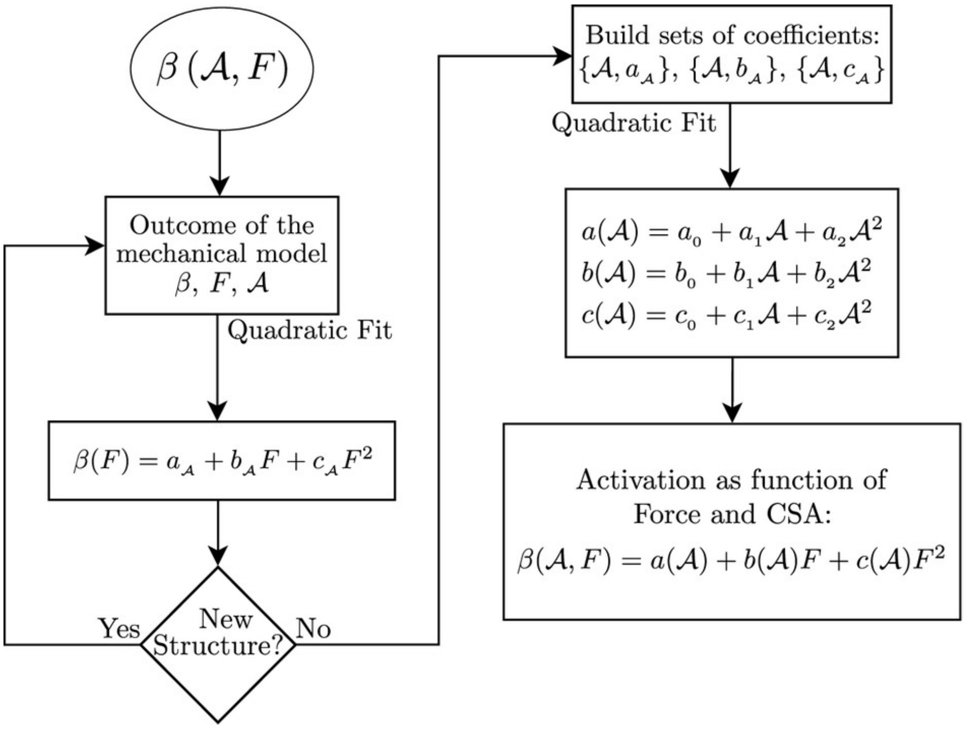

Figure S13. Procedure to calculate the activation level β required to produce a given force F with a given CSA $\mathcal{A}$ of the muscle structure.
